# AccessLab: Workshops to broaden access to scientific research

**DOI:** 10.1371/journal.pbio.3000258

**Published:** 2019-05-28

**Authors:** Amber G. F. Griffiths, Ivvet Modinou, Clio Heslop, Charlotte Brand, Aidan Weatherill, Kate Baker, Anna E. Hughes, Jen Lewis, Lee de Mora, Sara Mynott, Katherine E. Roberts, David J. Griffiths

**Affiliations:** 1 FoAM Kernow, Cornwall, United Kingdom; 2 British Science Association, London, United Kingdom; 3 College of Life and Environmental Sciences, University of Exeter, Penryn Campus, Cornwall, United Kingdom; 4 Centre for Water Systems, College of Engineering, Mathematics and Physical Sciences, University of Exeter, Exeter, United Kingdom; 5 Plymouth Marine Laboratory, Plymouth, United Kingdom

## Abstract

AccessLabs are workshops with two simultaneous motivations, achieved through direct citizen-scientist pairings: (1) to decentralise research skills so that a broader range of people are able to access/use scientific research, and (2) to expose science researchers to the difficulties of using their research as an outsider, creating new open access advocates. Five trial AccessLabs have taken place for policy makers, media/journalists, marine sector participants, community groups, and artists. The act of pairing science academics with local community members helps build understanding and trust between groups at a time when this relationship appears to be under increasing threat from different political and economic currents in society. Here, we outline the workshop motivations, format, and evaluation, with the aim that others can build on the methods developed.

## Abstract 2

This Community Page article describes AccessLabs: workshops involving direct citizen-scientist pairings, with the aims of decentralising research skills to a broader range of people and exposing science researchers to the difficulties of using their research as an outsider, creating new open access advocates.

## Introduction

Globally, there are roughly 33,100 peer-reviewed academic English-language research journals, responsible for publishing about 3 million articles a year, with the number of articles rising consistently by about 3% each year [1]. Estimates from CrossRef, Google Scholar, and the Web of Science indicate that in total, roughly 70 million articles have been published to date in academic journals [1]. This is clearly a vast and growing resource of humanity's knowledge. However, only about 28% of research literature is open access (meaning an article is free to access and read in full online), while the rest is locked behind paywalls and as such is unaffordable to many people. Although the proportion of freely available research papers is growing, even in the most recent year analysed (2015), only about 45% of new articles were found to be open access [2].

There is considerable demand for journal articles from people who do not work in wealthy institutions that are able to afford journal subscriptions. For example, we know from Sci-Hub records that around 400,000–600,000 pirated downloads of journal articles take place every day (data from late 2017 [3]). The Open Access Button (https://openaccessbutton.org/) collects the reasons why people request articles, and these frequently include academics and students in less wealthy countries/institutions, librarians, teachers, health professionals, and medical patients. The ability to read and use research articles is clearly of value for a wide range of people—it is easy to understand why a medical professional or patient might want access to the latest research, or how access to research articles might benefit private sector research and development progress [4]. Similarly, people in a broad range of jobs such as policy making, renewable energy, farming, city planning, or teaching could also find access to the latest research useful, and anyone making day-to-day decisions such as whether to vaccinate a child might benefit from access to reliable information.

There are currently few approaches that adequately address the issue of lack of equal access to scientific information. Tools exist that keep track of when paywalls are problematic (e.g., the Open Access Button), resources and organisations exist that summarise or translate scientific research for broader audiences (e.g., the Parliamentary Office of Science and Technology, Sense about Science), platforms exist for people to listen to or pose questions of scientists (e.g., Café Scientifique, Soapbox Science, Reddit’s r/askscience), and systems exist to provide research support in response to concerns raised by civil society (e.g., Science Shops). However, most of these approaches are unidirectional and the balance of power is maintained by the science academics (i.e., scientists provide answers), and none of these approaches pass on the skills necessary to enable people to find and judge research on topics that matter to them.

While surveys typically show that academic scientists are highly trusted, the same surveys tend to show that journalists are poorly trusted [5,6]. Indeed, the majority of people 'agree that the media sensationalises science' (71%) and that 'there is so much conflicting information about science it is difficult to know what to believe' (70%) [7]. When people are not able to access primary information in the form of research publications, they are left with more widely and freely available secondary sources of information, such as mass media coverage, social media, or publications by charities or corporations. It is worth noting that the authors of these secondary sources of information often do not themselves have direct access to the full primary sources. This poses a clear problem if people want to find information about science that they can trust but face barriers such as discoverability, accessibility, and affordability of papers from primary source research journals. This disparity in the type and quality of the sources of information that are available to people is known as knowledge inequality and is recognised by the United Nations Educational, Scientific and Cultural Organization (UNESCO), who state that 'knowledge inequalities between individuals and groups affect the capacity to make informed decisions, to access services and to participate in political life' [8].

Progress driven by open access advocates and innovators is likely to alleviate the biggest barrier to research accessibility to some degree in future. For example, Plan S requires that, from 2020, scientific publications resulting from research funded by public grants must be published in compliant open access journals or platforms (although this does nothing to improve access to publications pre-2020, https://www.coalition-s.org/). However, to make use of the research papers that become freely available, people need to know three fundamental things: (1) that research papers even exist, (2) how to get hold of them, and (3) how to read and interpret them. At this point in time, we believe it is essential to begin to think about how we can enable people to find and use research once it becomes more broadly available through the advances made by the open access movement.

### Overview of the AccessLab approach

AccessLab is a workshop format primarily designed to decentralise research skills to enable a broader range of people to access and use scientific research, reducing knowledge inequality. The workshops involve pairing people who want help accessing scientific research with local academic science researchers, to co-research a science-related question/topic of use to the participant who is not from an academic science background.

Without necessarily even being aware of it, academic science researchers possess the extremely valuable basic skills of knowing how to find sources of information and how to judge the reliability of that information. Through the act of co-research during AccessLab workshops, these skills in finding and judging information are passed on to others. To ensure that skills that are passed on rather than subject-specific knowledge, we make sure that researchers are paired with people who have questions that are outside the researcher's specialism. This approach means that the participants leave with a skill set that they can apply to researching other topics and questions in the future, rather than simply leaving with subject-specific answers to a single question. This offers a rare opportunity for people outside academia to learn about how scientific research actually works, rather than simply considering them as consumers of the end products of the research. Through this process, the academic science researchers realise the inherent value of their research skills and learn firsthand from their paired participant about why people from outside academia want to be able to read research papers. The researchers also experience (often for the first time) the difficulties of accessing their research as an outsider, creating new open access advocates.

The format directly challenges the power imbalances typical in public engagement practices by placing all participants on an equal footing, learning from each other, as opposed to the standard approach of didactic or performative events led by academics. Additionally, the workshops help to improve understanding and trust between different sectors, and connect local communities.

AccessLab was originally developed based on a pilot event in 2015, in which a small number of participants from mixed backgrounds were asked about the sources of information they would use to research a new medical diagnosis—there were clear differences, with science academics typically seeking peer-reviewed and primary sources, while others mainly used secondary sources. During this workshop, a pairing format was discussed as a solution—we then held two trial AccessLab workshops in 2017, first in Penryn in Cornwall for artists, and then in Redruth in Cornwall for councillors and community groups/activists. Modifications and improvements were made iteratively based on participant feedback, and write-ups from these earlier workshops outlining the problems and improvements are freely available online (https://fo.am/accesslab/). With a well-honed format, we then launched a series of three workshops in 2018, trialling the format with new audiences—firstly for the marine sector in Penzance, secondly for media/journalists in Exeter, and thirdly for policy makers in Plymouth. We prioritised Cornwall and Devon, as these are regions where few such events take place, and prioritised towns that ranked highly on the Indices of Multiple Deprivation (https://www.cornwall.gov.uk/council-and-democracy/data-and-research/data-by-topic/deprivation/).

Because the non-academic-science-researcher participants are from very varied backgrounds, for simplicity, from this point on we refer to this group as ‘citizen participants’. The structure outlined below represents the AccessLab format for the workshops held in 2018.

### AccessLab workshop structure

The first consideration for running an AccessLab is identifying a citizen participant group who might benefit from access to scientific research, and an appropriate geographical location (e.g., our marine sector workshop was run in Penzance due to the high density of marine-related organisations locally). The next task is to find a suitable venue—in our trials (with the exception of one event), we have avoided locations linked to formal learning (e.g., universities, schools, libraries) and aimed for as neutral a location as possible, offering plentiful space and a pleasant environment. We place great emphasis on ensuring a welcoming environment within the venues, for example, using flowers, cushions, and lighting to disrupt or remove pre-existing expectations of learning situations. Food is also treated as a primary rather than secondary feature, as when taken seriously it can help bring people together and make them feel valued. One participant commented that ‘all the flowers and little touches make all the difference–it meant I realised it wasn’t going to be a test and was just more at ease, I also thought it meant they actually wanted me there’.

Once a venue is chosen, we invite the people from the citizen participant group and academic science researchers who are based locally—this has the benefit of improving the participants' local networks, allowing the opportunity for longer-term collaborations to form. We cap the numbers at 16 (8 pairs) and offer financial support for travel/accommodation/childcare to all participants who might otherwise be unable to attend (with the exception of the first workshop). On signing up, the citizen participants are asked what science-related question or topic they want to look into during the workshop, and this is used for deciding on pairings. For example, a town councillor wanted to research the impacts of building developments on the environment, a general practitioner (doctor) wanted to research nutrition for advising patients with specific diseases, and a dancer/choreographer wanted to research physiology and injuries. The academic science researcher participants in the five AccessLab workshops were predominantly environmental scientists due to the focus of the research departments in the geographical region and the source of the funding for supporting the workshops (the final three were funded by the Natural Environment Research Council).

Each workshop consists of two sessions—a preparatory session just for the academic science researchers, and the main session for all participants. The workshops consist of facilitated tasks and short talks, which are outlined in detail in Boxes 1 and 2.

**Box 1. Details of the workshop format, day 1: Preparatory session for academic science researchers**

**Fig 1:**
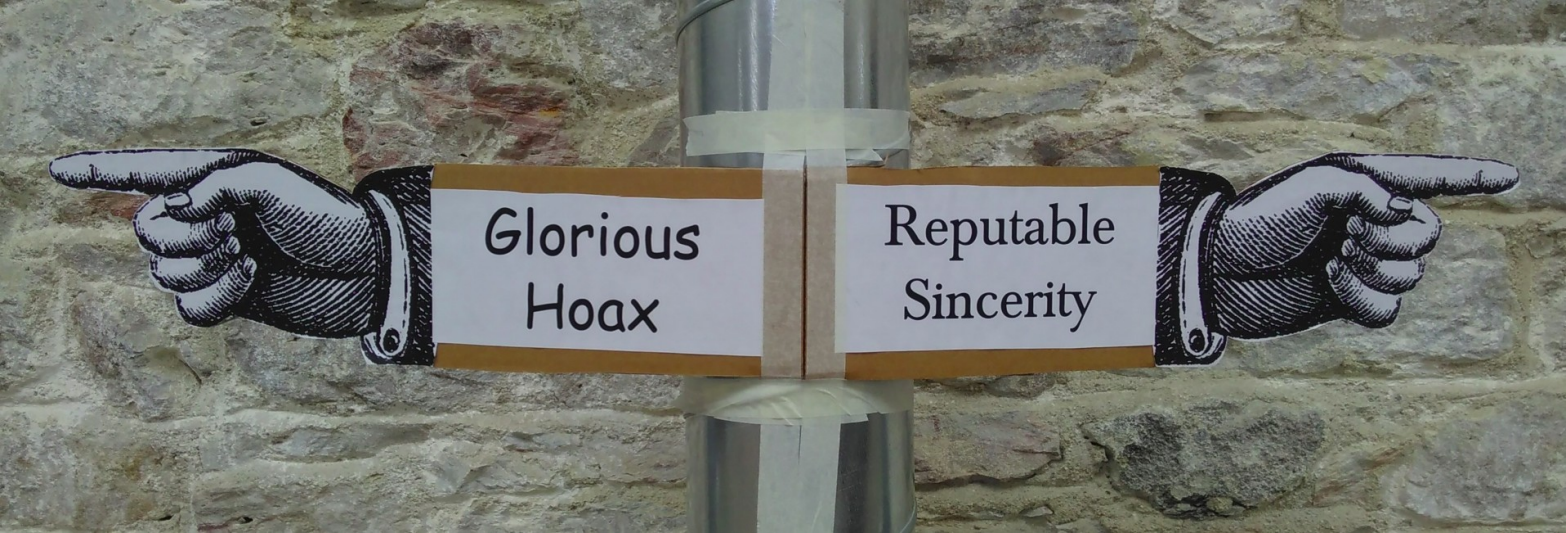
The corridor of trust. Participants rank the sources of information that they use along a scale from Glorious Hoax to Reputable Sincerity.

**Information Task 1** (30 minutes)—Participants are asked a question outside their expertise, e.g., ‘if there were a new referendum on UK membership to the EU, which sources of information would you use to decide how to vote’? Participants write each information source on a separate small piece of paper. We set up a ‘corridor of trust’, with a scale running from ‘Glorious Hoax’ at one end of a corridor to ‘Reputable Sincerity’ at the other (Fig 1). Participants are asked to place each of their sources of information on the scale, depending on how trustworthy they believe that information to be. Some critical points from the exercise are as follows: (i) science academics rarely use peer-reviewed journals to find out about subjects other than their own—we almost never see politics or economics journals listed—therefore, it is not sensible to assume that people from other backgrounds will be using scientific journals to get their information about science, and (ii) there are always plenty of sources of information down at the Glorious Hoax end of the scale—most people use sources of information that we already know we do not trust.

**Information Task 2** (20 minutes)—Participants are asked what they think the biggest problem of our time is, that scientific research has something to offer. We then pair up the researchers and give each pair one of the big issues. They then have 15 minutes to choose 3–5 of the most important research papers that they would want people to read about that topic. Each pair writes their topic and list of papers onto a large sheet of paper. Once they are completed, these are taken away. While the workshop continues, one of the facilitators stamps a red dollar sign on any that are paywalled. We bring the sheets back at the end of the workshop to show that many (typically approximately 50%) of the articles that the researchers suggested as the most important for people to read are inaccessible to anyone without an institutional subscription/log-in.

**Information Task 3** (30 minutes)—We ask participants two things: (i) how do you find and access scientific information, and (ii) how do you judge the quality/reliability of a scientific research paper. Participants are asked to put their answers on individual small pieces of paper. We have two big charts set out on the floor, one for each of the two questions. The x-axes run from ‘easy’ to ‘difficult’, referring to how easy/difficult it would be for someone without a background in academia to use these methods. The y-axes run from ‘works well’ to ‘doesn’t work well’, referring to how good the method is, either for finding papers or judging their reliability. This makes the participants think about how accessible the methods that they use are for other groups. We then ask the group to vote on the best and worst methods for using with a non-science-academic audience.

**Virtual private network (VPN) check** (5 minutes)—We provide participants with two links to academic papers that are behind paywalls and ask them to use their own laptops to see if they can access the papers. If they can, we know that they have VPNs or institutional journal log-ins set up, and we ask them to switch these off before the main workshop, so that they are researching with the same resources that everyone else has access to.

**Box 2. Details of the workshop format, day 2: Main workshop for academic science researchers and chosen audience group**

**Information Task 1** (30 minutes)—Performed as for task 1 above; however, this time participants are asked a question that is related to science—for example, ‘if you wanted to look into the problem of marine plastics, and what you could do about it, what sources of information would you use’? Some critical points that we make from the exercise are as follows: (i) Splits appear in the sources of information that each group uses. Typically, the researchers use peer-reviewed journals (because they are now within their field of expertise), while the citizen participant groups make more use of charities and nongovernmental organizations (NGOs). Our aim is to demonstrate to the citizen participant group that they could make use of peer-reviewed journals, and also to show the academic science researcher participants where they might want to disseminate their work if they want people to read/use it. (ii) Academic science researchers typically list colleagues and research institutions as sources of information, while the citizen participant group usually don’t have these types of contacts. We point out that this is a form of privilege—but also that the workshop is a chance to make this type of contact.

**Introductory talk and discussion** (70 minutes)—Here, we give three separate talks to provide necessary context: firstly, an introduction to the AccessLab project, mentioning the value and quantity of scientific information that exists, and exposing participants to the current lack of accessibility to this information. In this section, we talk about the history of academic publishing and how the current situation arose (15 minutes) (inspired by [9]). Secondly, we provide an introduction to how scientific research is funded in the UK, and how researchers choose what to work on (10 minutes). Thirdly, we provide an introduction to how scientific publishing works—what journals are, what review papers are, and how peer review takes place (10 minutes). Finally, there is time (about 35 mins) for questions arising from the introduction. Here, we encourage people to answer each others’ questions. The talk slides from the final event are available here: https://figshare.com/articles/AccessLab_-_main_event_intro/7364990.

**Media case study** (15 minutes)—A talk covering how research ends up in the media, then taking a media article and tracing it back to the original research, providing straightforward steps that people can take to judge the reliability of the work: (i) can you find the original research, (ii) who did the work, (iii) who funded the work, (iv) how was the work done (what are the sample sizes; was the research actually done on humans if the media article is making statements about humans; if it is a review, does it use the words ‘systematic review’), (v) did the researchers actually do what the media article says they did, (vi) what have others published previously/since, and (vii) can you access the data. The talk slides are available here: https://figshare.com/articles/AccessLab_-_media_case_study_-_Penzance_2018/6893870.

**Break for lunch** (60 minutes)—We serve a full lunch, either self-catered or provided by local suppliers. This is a good opportunity to support the local area by hiring an up-and-coming chef.

**Media case study pairings** (30 minutes)—We pair people up with their neighbours (participants are seated alternating between academic science researchers and other participants, but at this stage people are not seated next to the person they will be paired with for the main co-research session) and provide each pair with a science-related media article to scrutinise following the steps they have learned. At the end, a short group discussion means participants can say if they think their media article is reliable or not, and share additional tips for fact-checking.

**Co-research in pairs** (120 minutes)—The pairs of academic science researchers and citizen participants are arranged in advance, with researchers placed with people who have questions outside the researcher's specialism. The exception to this rule is that when the citizen participant has a social science question, we will pair them with a social scientist, as the research methods are quite different.

The pairs are given two hours to do research together on the topic/question that the citizen participant came with. We suggest that the first 30 minutes are spent on honing the question, as formulating the right question is a big part of doing good research. Otherwise, there is no structure to this session, and the pairs are free to work as they like. There is no expectation that the participants report their work to the group, and this section is considered confidential within the pairs.

### Data analysis and evaluation

#### Comparing the sources of information used by each group

We gathered the sources of information from Information Task 1 on days 1 and 2 at each workshop (available in S1 Data). The type of information sources listed by the academic science researchers were very different depending on whether they were asked a question that related to science or not. When asked a political question, the academic science researchers rarely used primary information (e.g., politics journals or governmental data), instead favouring mass media, but when asked a scientific question, they overwhelmingly relied on primary information (e.g., scientific journals).

When asked a science-related question, the citizen participant groups listed a smaller number of resources than the academic science researchers in all five workshops, and overwhelmingly relied on environmental charities/NGOs as a source of scientific information, with few mentioning primary sources of information, like scientific journals.

### Analysis of researchers' suggested methods for finding, accessing, and judging information

We gathered the papers from Information Task 3 on day 1 at each workshop (available in S2 Data). When asked about where people might be able to find and access scientific information, the most common suggestion was Google Scholar. Other suggestions that came up frequently were Web of Knowledge/Web of Science, Twitter via #icanhazpdf, ResearchGate, looking on the authors' websites, emailing the author, arXiv preprints, and PubMed. Many of the suggestions for where people might be able to access scientific information required implicit knowledge gained through working within the academic system (e.g., the possibility of emailing authors and using the Twitter hashtag #icanhazpdf are unlikely to be widely known outside academia) or the use of systems that are targeted firmly at academics (e.g., ResearchGate), are primarily discovery tools (as opposed to accessibility tools), and/or require an institutional subscription (e.g., Web of Knowledge). Aside from Google Scholar, remarkably few science researchers suggested systems that are more accessible outside academia, such as Sci-Hub (2 mentions out of 22 researchers, although this may be lower than expected if researchers were wary of mentioning it because of it being a pirating site), and legal options (primarily search engines optimised for academic information, or peer-to-peer sharing approaches) such as Unpaywall (one mention), Kopernio (no mentions), Open Access Button (no mentions), Reddit Scholar (no mentions), or the upcoming search engine Get the Research (no mentions). This session revealed quite limited knowledge and understanding of discoverability and accessibility tools within the academic community but provided a valuable opportunity to learn how best to help others find and use academic research.

When asked about methods for judging the reliability of scientific information, the most common suggestion was to look at how many times the paper had been cited, followed closely by looking at whether the authors had used robust methodology in their research. Other suggestions that came up frequently were to consider the journal reputation, author affiliations, journal impact factor, personal familiarity with the authors' work or reputation, and the funding source for the research. Many of the suggestions for how to judge the reliability of scientific information rely on research expertise (e.g., judging the methodology), subjective assessments based on experience obtained within academia (e.g., the authors' or journal's reputation), metrics that can be severely misleading (e.g., how often a paper is cited—for example, a well-known paper linking vaccination to autism is widely known within the scientific community to be fraudulent, but has a high citation number, as many of the citations are negative), or metrics that are proven to be inversely related to quality (e.g., journal impact factor [10,11]). Some suggestions were more suitable for those with no experience in scientific research, for example, looking at who funded the research to check for obvious conflicts of interest—appropriate methods were demonstrated on day 2 in the media case study.

‘I take a lot for granted when doing research—access to journals, how to read a paper, knowing "good" and "bad" methods…’—Researcher participant, Exeter 2018

#### Feedback and evaluation

For the first two AccessLab workshops in 2017, feedback was unformalised—for the three workshops in 2018, we gathered more formal feedback, which is presented below, in brief, and in detail in S1 Text. All evaluation data were collected and analysed anonymously, with the exception of the long-term feedback, which was emailed and then anonymised. The feedback forms used are available here: https://figshare.com/articles/AccessLab_-_Feedback_forms_-_Plymouth_2018/7370552, and feedback is available in full in S3 Data.

The evaluation data indicated that the AccessLab format is reasonably successful at reaching groups that are not particularly connected with science, achieves the aim of improving confidence and awareness of research methods for a broad range of people, and improves participants’ confidence in collaborating across sectors. Based on more limited data, the workshops appear to attract researchers that are already open science advocates and strengthen their pre-existing values.

**Fig 2:**
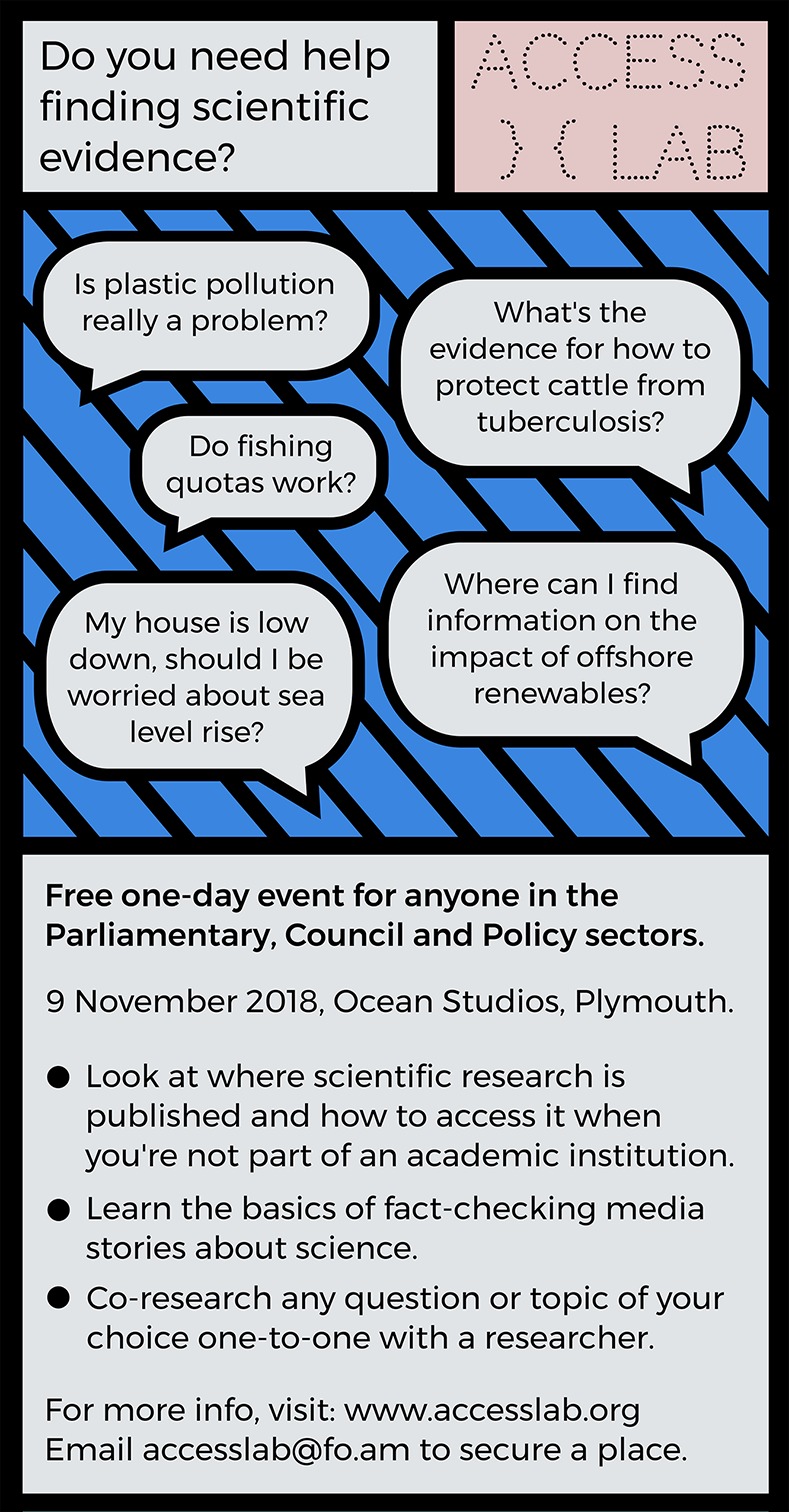
The advertisements for AccessLab gave participants prompts to see what they might get out of the day. **Free-text feedback from participants indicated that the aims of the workshop were met**. ‘It’s brilliant… it’s a long time since I was part of a workshop that gave me so much in just one day. I feel I have fundamentally shifted the bounds of possibility.’—Policy sector participant, Plymouth 2018. ‘Confidence that researchers can be useful to other sectors in very direct ways! Plus to stand up for my open access principles.’—Researcher participant, Plymouth 2018. ‘I want to interact much more with local organisations/government. I realise we have much we could accomplish together, even if not directly related to my research.’—Researcher participant, Plymouth 2018.

When asked to rate the importance of each section of the workshops (in order to see if we could remove any section to shorten the day), no section of the workshops was ranked lower than 'Fairly Important' or 'Very Important'. The four workshop sections that were ranked by the majority as 'Very Important' were (i) research in pairs—answering the question, (ii) research in pairs—honing the question, (iii) fact-checking a media story together, and (iv) where to find scientific information/judge it. Free-text comments also indicated that the aims of the workshop were met (see Fig 2 for examples), and are available in full in S3 Data.

**Fig 3:**
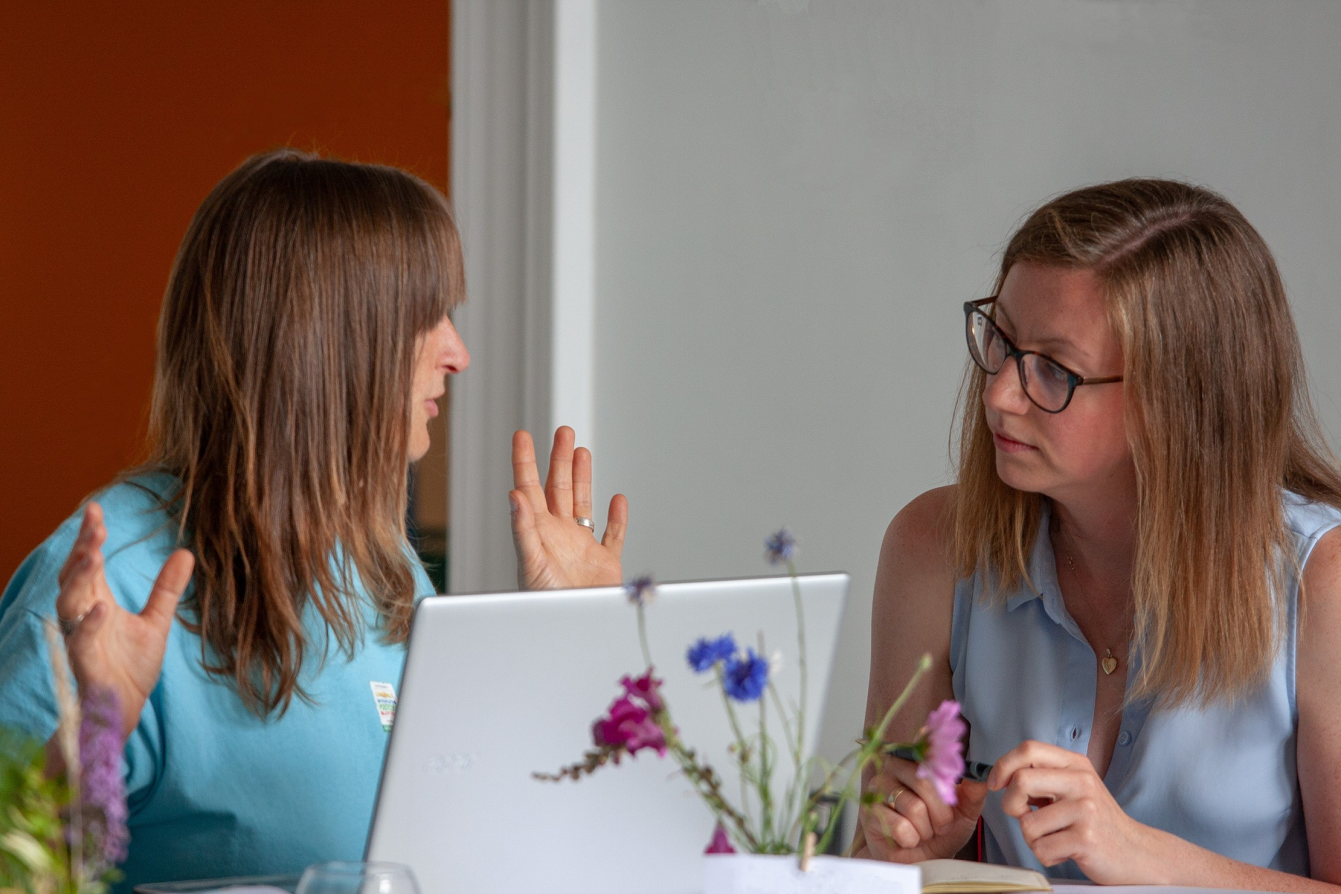
Feedback from participants indicated broad-ranging long-term benefits. ‘I found the AccessLab workshop really useful—it has helped me search for fisheries related projects/research, which I have then taken forward as examples to support investigations which I am currently working on. Delighted to be able to pull a more scientific strand into my investigations.’—Marine sector participant, Penzance 2018. ‘It has definitely made me think differently about academic publishing and how to get my research out there, which I hope will release me from what has been a major (potentially imagined) block to communication.’—Council/Community group participant, Redruth 2017. ‘Working with artists during AccessLab has given me more confidence approaching artists about collaborative work. Primarily, I think, because it gave me a better sense of an artist's perspective. What they might be looking for in a project, a better understanding of their unique approach and the importance of having such a different skill set.’—Researcher participant, Penryn 2017.

We followed up participants 2–4 weeks and 5–6 months after each event—this longer-term feedback indicated that some participants had stayed in touch, developed new projects together or with contacts gained through the workshops, changed their working practices, and even started working on a joint publication (see Fig 3 for examples). A full case study is available in S2 Text, describing a policy change that directly resulted from co-research during an AccessLab.

### The future of AccessLab

We hope that the approaches outlined in this paper will be used by others in their own events (noting that the format is Creative Commons Attribution-ShareAlike licenced); however, the future of AccessLab remains open at this stage. After the five AccessLabs, we brought participants back together for a scoping workshop. When given a choice of nine possible future scenarios for the AccessLab project, the most popular scenario amongst our participants was to allow people to run their own workshops under the AccessLab name, but to require the workshop leader to have participated in an AccessLab previously (see S4 Data for scenarios presented and votes on each). This approach would allow the project to roll out gradually, without the need for centralised organisation, and would help to retain the ethos of the project. There is, of course, a risk with this approach that the ethos may gradually be lost, and suggestions to alleviate this risk were to formulate a centralised 'AccessLab manifesto' and a resource of frequently asked questions. Although many of our participants stayed in touch after the workshops, it may also be useful to build more formal encouragement of longer-term relationship building into the format, for example, through repeat events or facilitating the participants to visit each other's workplaces.

Throughout the five AccessLab workshops, it was easy to recruit academic science researchers to participate—there is a clear appetite for new approaches to public engagement both from researchers and funders. Indeed, AccessLabs are suitable for inclusion in grant applications (for example, as 'pathways to impact' for UK research council applications) and also potentially for inclusion in university research quality assessments (for example, as impact case studies in the Research Excellence Framework in the UK). Feedback from participants indicated that AccessLab helped researchers to learn how to make their work relevant and accessible for stakeholders, which fits well with the impact agenda currently championed by many funders.

Generally speaking, it took more effort to recruit participants from nonacademic sectors, and the ease depended on the strength of our own personal networks in each sector. A popular suggestion amongst participants at our scoping workshop was that future AccessLabs could be run by duos, consisting of one academic science researcher and one contact from another sector—this would make it easier to find participants in the other sector and help to ensure the element of equality between the two participant groups is retained.

## Conclusions

The Open Access publishing movement continues to grow—fully open access megajournals like *PLoS One* and *PeerJ*, platforms such as F1000Research and ScienceOpen, the launch of university press journals like *UCL Open*: *Environment*, self-archiving in searchable centralised repositories like arXiv or those produced using the OSFpreprints platform—all offer considerable promise for the accessibility of future research, as do policy movements like Plan S and major shifts in research funders’ policy decisions (e.g., United Kingdom Research and Innovation https://www.ukri.org/funding/information-for-award-holders/open-access/). However, great challenges remain, and two are particularly important: firstly, securing legal access to publications that have already been published behind a paywall, and secondly, beginning to shift the discourse to how we enable and encourage people from broad walks of life to make use of the newly accessible research. More generally, we see AccessLab as having the potential to enhance mutual understanding and trust between academic scientists and the people who pay for their work at a time when this relationship may become under threat from different political and economic currents in society. We hope that the AccessLab workshop approach provides a starting point, allowing others to continue to develop and improve on the methods trialled.

## Supporting information

S1 Data Sources of information listed by participants. (XLSX)

S2 Data Researcher participants' suggested methods for finding/judging information. (XLSX)

S3 Data Evaluation and feedback—Raw data. (XLSX)

S4 Data Future scenarios and voting. (XLSX)

S1 Text Evaluation and feedback—Detailed report. (DOCX)

S2 Text Case study: Evidencing idling coaches in Dartmouth. (DOCX)

## Abbreviations

NGO: nongovernmental organization
UNESCO: United Nations Educational, Scientific and Cultural Organization
VPN: virtual private network
