# Supplementary material for: AccessLab: Workshops to broaden access to scientific research
Source: PLoS Biol. 2019 May 28;17(5):e3000258. doi: 10.1371/journal.pbio.3000258 (PMC6538137; doi:10.1371/journal.pbio.3000258)
Supplement: S1 Text — (DOCX) [file pbio.3000258.s005.docx]

**S1 Text. Evaluation and feedback - detailed report**

Feedback and evaluation forms used are available in full here: <https://figshare.com/articles/AccessLab_-_Feedback_forms_-_Plymouth_2018/7370552>.

At each of the three workshops in 2018, at the start of day 2 we asked the citizen participants (i) about their relationship with science (4 options: I work in a science related job or study a science subject/I feel connected with science – I actively seek out science news, events, activities or entertainment/I’m interested in science but I don’t make a special effort to keep informed/Science is not for me) and (ii) to rank ‘How confident do you feel researching scientific questions/topics’ and ‘How confident do you feel working with the other participant group (as opposed to your usual colleagues/peer groups)?’ (5 options: Not at all confident/Not very confident/Neutral/Quite confident/Very confident). Similarly, we ask the academic science researchers to (i) rank ‘How confident do you feel working with the other participant group (as opposed to your usual colleagues/peer groups)?’ and (ii) ‘How important do you think it is to publish your own research Open Access?’ (this final question was only asked in the last workshop), using the same 5-point scale. These questions were revisited at the end of the day and changes in rankings were marked by the participants.

Of the 18 non-academic-researcher participants who completed the information about their relationship with science, 9 ranked themselves as 'I’m interested in science but I don’t make a special effort to keep informed', 2 ranked themselves as 'I feel connected with science – I actively seek out science news, events, activities or entertainment', and 2 ranked themselves between levels. This indicates that the AccessLab format is reasonably successful at reaching groups that are not particularly connected with science, but as anticipated, does not reach those who rank themselves as 'science is not for me'.

When asked 'How confident do you feel researching scientific questions/topics?', the mean rank change for the non-science-academic groups was +1.61 (n=18), indicating that the workshop format achieves the aim of improving confidence and awareness of research methods for a broad range of people. We also saw improvements in confidence collaborating across sectors – when asked 'How confident do you feel working with the other participant group (as opposed to your usual colleagues/peer group)?', the mean rank change for the citizen participants was +1.29 (n=17), and for the academic science researcher participants was +0.86 (n=22). When asked 'How important do you think it is to publish your own research Open Access?', the mean rank change for the academic-science-researchers was +0.5 (n=8). This question was only asked at the final workshop, and all academic science researcher participants ranked the importance of Open Access as either quite or very important at the start of the workshop, so the workshop format appears to attract researchers that are already open science advocates and strengthen their pre-existing values.

For the three 2018 workshops we also asked all participants to rate the importance of each section of the workshops (in order to see if we could remove any section to shorten the day), and to fill out three free-text questions: (1) What is the most useful thing you have learned from AccessLab? (ii) How has AccessLab made you think differently about your local area? (iii) Is there anything else you would like to tell us about AccessLab? Participants were also given a chance to give verbal feedback as a group at the end of the workshops, and we followed up participants 2-4 weeks and 5-6 months after each event to ask for any free comments on whether coming to AccessLab changed anything they do.

On average, across all workshops, no section of the workshops was ranked lower than 'Fairly Important' or 'Very Important'. The four workshop sections which were ranked by the majority as 'Very Important' were (i) research in pairs – answering the question, (ii) research in pairs – honing the question, (ii) fact checking a media story together, (iii) where to find scientific info/judge it. Free-text comments indicated that the aims of the workshop were met (see Fig 2 for examples), and are available in full in S3 Data.
