# Supplementary material for: AccessLab: Workshops to broaden access to scientific research
Source: PLoS Biol. 2019 May 28;17(5):e3000258. doi: 10.1371/journal.pbio.3000258 (PMC6538137; doi:10.1371/journal.pbio.3000258)

**S2 Text. Case study: Evidencing idling coaches in Dartmouth**

Dr Lee de Mora, Plymouth Marine Laboratory

Tessa de Galleani, Dartmouth Town Council

Tessa de Galleani, a town councillor from Dartmouth, came to the 5th AccessLab event in Plymouth with a specific problem that she wanted to address with the help of a scientist.

Dartmouth is a small and beautiful town on the West bank of the River Dart in South Devon. In the summer months, Dartmouth is a particularly popular tourist destination and many tour coach companies make a stop on their tour of South Devon. The coach parking area is along on The Embankment road, along the river bank in the town centre. On any given summer day, there are typically between 8-15 coaches parked to drop and collect their passengers for up to 20 mins at a time. Unfortunately, the coaches invariably leave their engines running while parked. The coach drivers declined to turn off their engines because they didn't want to turn off their air conditioning. Similarly, Tessa wanted to convince the District and Town Councillors that this was a meaningful problem. Near the coach parking, there are several park benches and a riverside cafe that are subjected to concentrated exhaust fumes and diesel particulates pollution.

At the AccessLab event, Tessa was paired with Dr. Lee de Mora, a marine ecosystem modeller from Plymouth Marine Laboratory. Over the course of the AccessLab, Tessa and Lee did some collaborative research and found several resources about idling diesel engines, news articles from various towns in the UK that have implemented idling fines, and some scientific papers about the links between air pollution and deaths in the UK. In addition, Lee produced an estimate of the total CO_2_ emitted by the coaches while idling (approximately 4Kg of CO_2_ per hour per vehicle).

With this new evidence, Tessa went back to Dartmouth Council and convinced the County, District and Dartmouth councillors. Permissions are now in place with full funding to put up several signs along Embankment asking drivers to stop their engines idling. In addition, the Dartmouth police sergeant agreed to help and is now asking coach drivers to turn off their engines. We estimate that this effort could reduce the amount of CO_2_ emitted in Dartmouth from idling coaches by up to 15 Tonnes of CO_2_ per summer.


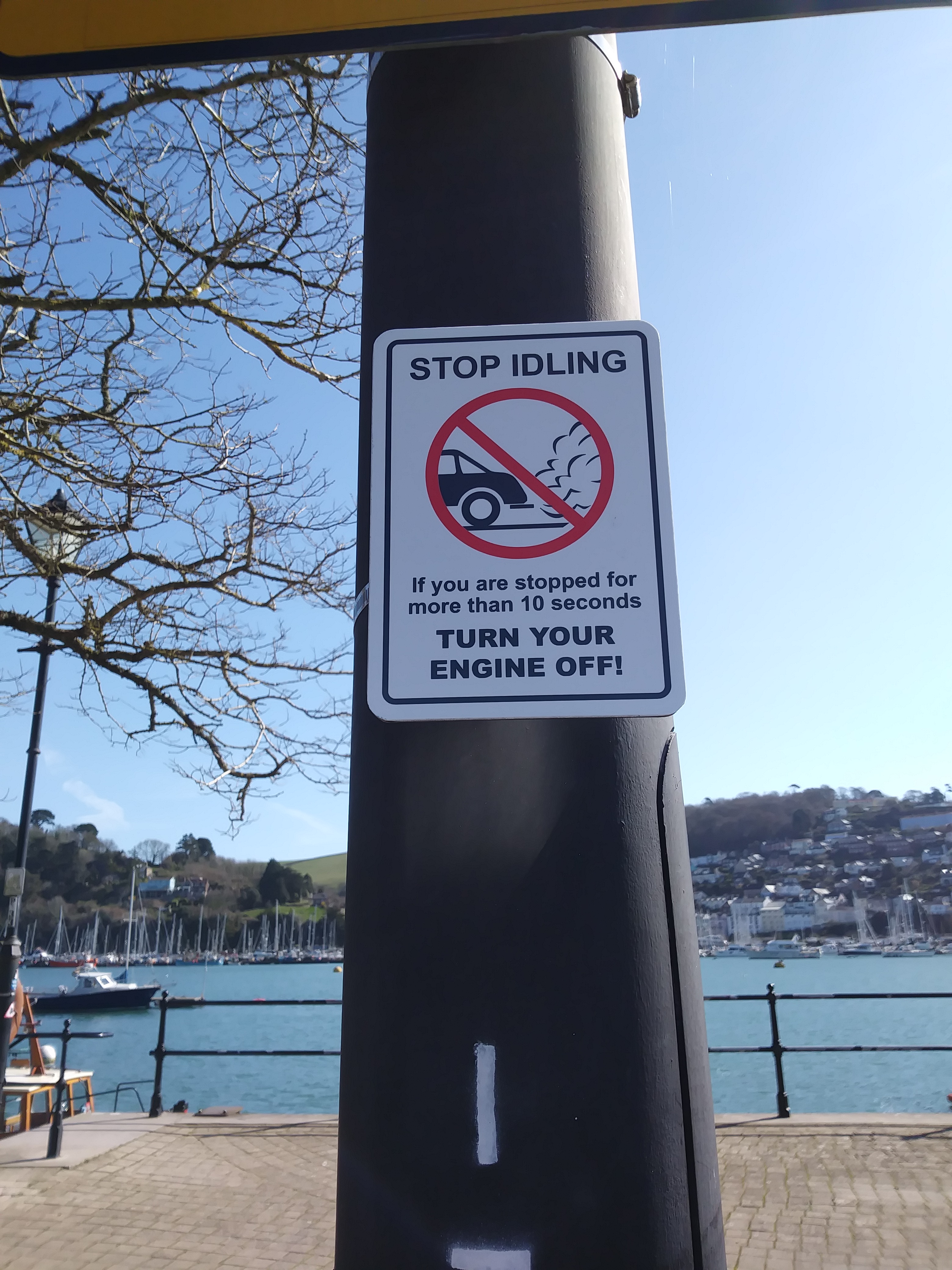

Supplement: S2 Text — (DOCX) [file pbio.3000258.s006.docx]
